# Gut Microbiota-Derived Propionic Acid Mediates ApoA-I-Induced Amelioration of MASLD via Activation of GPR43–Ca^2+^–CAMKII–ATGL Hepatic Lipolysis

**DOI:** 10.3390/ijms27010468

**Published:** 2026-01-01

**Authors:** Mengyuan Liu, Yutong Wang, Haixia Huang

**Affiliations:** 1 Department of Physiology and Pathophysiology, School of Basic Medical Sciences, Capital Medical University, Beijing 100069, China; lmyuan1997@163.com; 2 Laboratory for Clinical Medicine, Capital Medical University, Beijing 100069, China; 3 Department of Cell Biology, School of Basic Medical Sciences, Capital Medical University, Beijing 100069, China

**Keywords:** apolipoprotein A-I, gut microbiota, propionic acid, lipolysis, metabolic dysfunction-associated steatotic liver disease

## Abstract

Metabolic dysfunction-associated steatotic liver disease (MASLD) is a widespread hepatic condition characterised by hepatic lipid accumulation and inflammation. Emerging research highlights the contribution of the intestinal microbiota and its metabolic byproducts to the pathogenesis of MASLD through the gut–liver axis. Apolipoprotein A-I (apoA-I), the principal structural component of high-density lipoprotein (HDL), is linked to various metabolic disorders; however, its function in MASLD has not yet been clearly elucidated. This study sought to examine whether apoA-I protects against MASLD, with a focus on the possible role of the gut microbiota and propionic acid (PPA). The contribution of the gut microbiota was evaluated using faecal microbiota transplantation (FMT) and antibiotic cocktail (ABX)-mediated depletion. Microbial composition was assessed via 16S rRNA sequencing, and concentrations of short-chain fatty acids (SCFAs) were quantified. The effects of PPA on MASLD were examined using in vivo and in vitro models. The results showed that apoA-I overexpression alleviated MASLD in a gut microbiota-dependent manner, restored microbial homeostasis, and elevated PPA levels. PPA supplementation improved MASLD phenotypes. Mechanistically, PPA treatment was associated with the activation of the GPR43–Ca^2+^–CAMKII–ATGL pathway, suggesting that PPA plays a role in stimulating hepatic lipolysis and enhancing mitochondrial β-oxidation. These findings reveal a novel pathway through which apoA-I ameliorates MASLD by modulating the gut microbiota and increasing PPA levels, which activate a hepatic lipolysis cascade. The apoA-I–microbiota–PPA axis represents a promising therapeutic target for MASLD intervention.

## 1. Introduction

Metabolic dysfunction-associated steatotic liver disease (MASLD), a common chronic liver condition, affects more than 30% of patients worldwide [1]. The disease process starts with pathological accumulation of lipids within hepatocytes and can progress through multiple stages [2], including metabolic dysfunction-associated steatohepatitis (MASH), which is characterised by hepatocellular ballooning and inflammatory infiltration [3,4]. In a substantial subset of patients (12–40%), MASLD progresses to MASH, which, without effective treatment, may lead sequentially to fibrosis, cirrhosis, and hepatocellular carcinoma (HCC) [3,4]. The “multiple-hit” hypothesis remains the predominant framework for understanding MASLD pathogenesis [5], incorporating factors such as unhealthy dietary patterns, genetic and epigenetic factors [6], insulin resistance [7], and intestinal dysbiosis [8].

Growing evidence underscores the critical influence of the intestinal microbiota and its metabolic byproducts on the progression of MASLD via the gut–liver axis [8,9,10]. In particular, short-chain fatty acids (SCFAs), notably acetic acid, propionic acid, and butyric acid, are key metabolites derived from the fermentation of dietary fibre [11]. These SCFAs exert hepatoprotective effects in MASLD, notably by mitigating hepatic steatosis and inflammation [12,13,14,15,16]. Propionic acid (PPA), for example, enhances hepatic lipid oxidation and suppresses de novo lipogenesis, with recent work showing that PPA derived from *Megamonas funiformis* can ameliorate MASLD through activation of the AMPK–PPARα pathway [15]. Similarly, butyric acid regulates lipid metabolism through mechanisms involving the GPR41/43 signalling cascade [16].

Apolipoprotein A-I (apoA-I), which is the major protein of high-density lipoprotein (HDL), is important for facilitating reverse cholesterol transport (RCT) [17] and is known to mitigate atherosclerosis, inflammation, and diabetes-related metabolic dysfunction [18]. Previous investigations, including those from our group, have shown that apoA-I overexpression alleviates hepatic steatosis, improves insulin resistance, enhances autophagy, and reduces oxidative stress in models of MASLD. Specifically, apoA-I enhances insulin sensitivity through the PPARα pathway [19] and promotes liver autophagy via AMPK activation [20]. Additionally, the expression of COX-2 in hepatocytes is suppressed by apoA-I through a reduction in reactive oxygen species, further supporting its multifaceted hepatoprotective role [21].

It remains to be elucidated whether the protective actions of apoA-I against MASLD involve the regulation of the intestinal microbiota and associated metabolic byproducts. Accordingly, this study sought to determine whether apoA-I alleviates MASLD through gut microbiota modulation, with a particular focus on the microbial metabolite PPA. We further aimed to explore the molecular mechanism through which PPA promotes hepatic lipolysis and fatty acid oxidation, specifically by investigating the potential involvement of the GPR43–Ca^2^⁺–CaMKII–ATGL signalling cascade, and to assess the therapeutic potential of PPA supplementation in the progression of MASLD.

## 2. Results

### 2.1. ApoA-I Alleviates MASLD in Mice Induced by HFCFD

**Figure 1:**
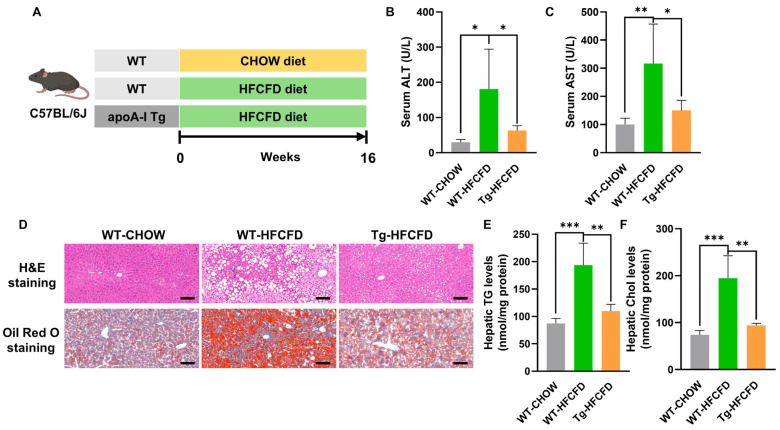
ApoA-I alleviates MASLD in mice induced by HFCFD. (**A**) Experimental design of the HFCFD-induced MASLD model. Male WT and apoA-I Tg mice, aged six weeks, were maintained on either a control chow diet or an HFCFD regimen for 16 weeks to assess apoA-I’s impact on MASLD progression. Serum concentrations of (**B**) ALT and (**C**) AST were measured. (**D**) Histological analysis of liver samples was conducted using H&E and Oil Red O staining (scale bar = 100 μm). Hepatic content of (**E**) triglycerides (TG) and (**F**) cholesterol (Chol) was quantified. Groups are color-coded: WT-CHOW (gray), WT-HFCFD (green), and Tg-HFCFD (orange). Corresponding group labels are shown on the x-axis. Data are represented as mean ± SEM; *n* = 5 mice per group. * *p* < 0.05, ** *p* < 0.01 and *** *p* < 0.001.

To assess the impact of apoA-I on MASLD development, we established a mouse model of MASLD using a high-fat, high-cholesterol, high-fructose diet (HFCFD) (Figure 1A and S1A,B). Compared with those in WT mice fed HFCFD, serum concentrations of alanine transaminase (ALT) and aspartate transferase (AST) were significantly lower in apoA-I Tg-HFCFD mice, indicating that elevated apoA-I mitigated hepatic injury in this model (Figure 1B,C; Table A1). The examination of liver sections stained with H&E and Oil Red O indicated that apoA-I significantly diminished HFCFD-induced hepatic steatosis and inflammatory infiltration (Figure 1D; Figure S2B–E). Furthermore, apoA-I significantly reduced hepatic triglyceride (TG) and cholesterol (Chol) levels in MASLD mice (Figure 1E,F). These results collectively suggest that apoA-I has therapeutic effects on MASLD.

### 2.2. ApoA-I Alleviates MASLD in a Gut Microbiota-Dependent Manner

**Figure 2:**
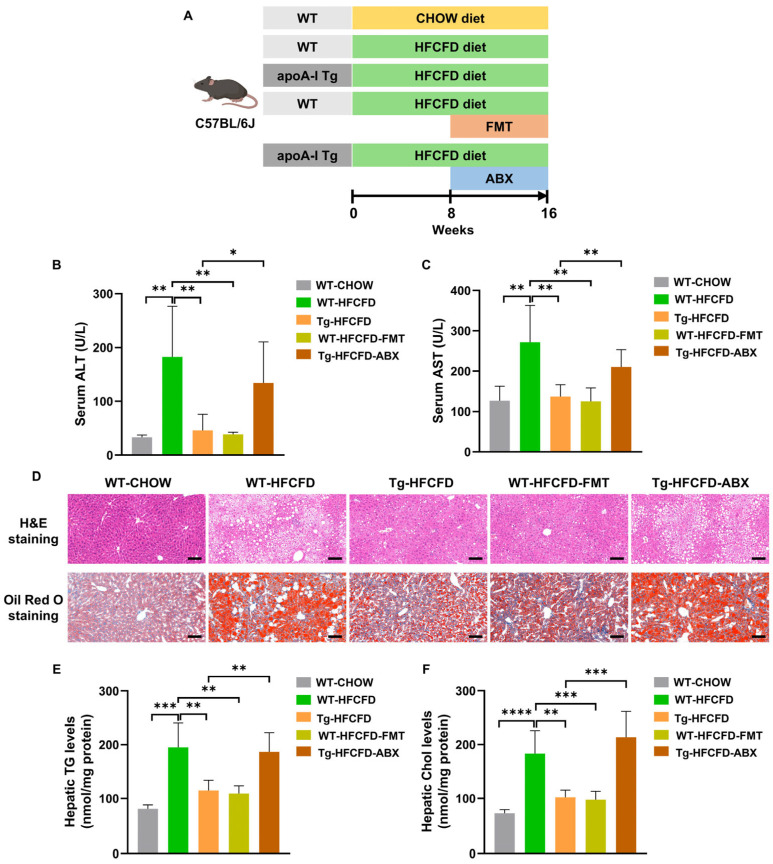
ApoA-I alleviates MASLD in a gut microbiota-dependent manner. (**A**) Male WT and apoA-I Tg mice, aged six weeks, were allocated into five experimental groups: WT-CHOW, WT-HFCFD, Tg-HFCFD, WT-HFCFD-FMT, and Tg-HFCFD-ABX. All animals were maintained on either a control diet or an HFCFD for 16 weeks. In the WT-HFCFD-FMT group, faecal microbiota transplantation (FMT) from Tg-HFCFD donors was administered over an 8-week period. Conversely, Tg-HFCFD-ABX mice were provided with an antibiotic mixture in their drinking water for 8 weeks to eliminate gut microbiota. Serum concentrations of (**B**) ALT and (**C**) AST were quantified. (**D**) Liver sections were stained via H&E and Oil Red O (scale bar = 100 μm). Levels of (**E**) hepatic TG and (**F**) Chol were evaluated. Groups are color-coded: WT-CHOW (gray), WT-HFCFD (green), Tg-HFCFD (orange), WT-HFCFD-FMT (lime green) and Tg-HFCFD-ABX (brown). Corresponding group labels are shown on the x-axis. Data are shown as mean ± SEM; *n* = 5 mice per group. * *p* < 0.05, ** *p* < 0.01, *** *p* < 0.001 and **** *p* < 0.0001.

Considering the well-documented involvement of the gut microbiota in MASLD pathogenesis [22,23], we investigated whether the protective effects of apoA-I on MASLD rely on these microbial communities. We conducted faecal microbiota transplantation (FMT) from HFCFD-fed apoA-I Tg mice (Tg-HFCFD) to HFCFD-fed WT recipients (designated WT-HFCFD-FMT). Additionally, we administered an antibiotic cocktail (ABX) to HFCFD-fed apoA-I Tg mice to deplete the gut microbiota (designated Tg-HFCFD-ABX) (Figure 2A; Figure S1C,D).

Compared with WT-HFCFD mice, WT-HFCFD-FMT mice presented significantly lower serum ALT and AST levels, hepatic steatosis, hepatocellular ballooning, and hepatic TG and Chol contents (Figure 2B–F; Figure S2G–J; Table A2). These findings indicate that FMT from apoA-I Tg mice effectively mitigates MASLD. Conversely, compared with Tg-HFCFD mice, Tg-HFCFD-ABX mice exhibited exacerbated hepatic injury, steatosis, and ballooning (Figure 2B–F; Figure S2G–J; Table A2). These findings indicate that the depletion of intestinal flora eliminates the hepatoprotective benefits of apoA-I in the context of MASLD. Collectively, our observations strongly support the conclusion that apoA-I alleviates MASLD in a gut microbiota-dependent manner.

### 2.3. ApoA-I Modulates the Gut Microbiota Composition and Increases the PPA Level in MASLD Mice

**Figure 3:**
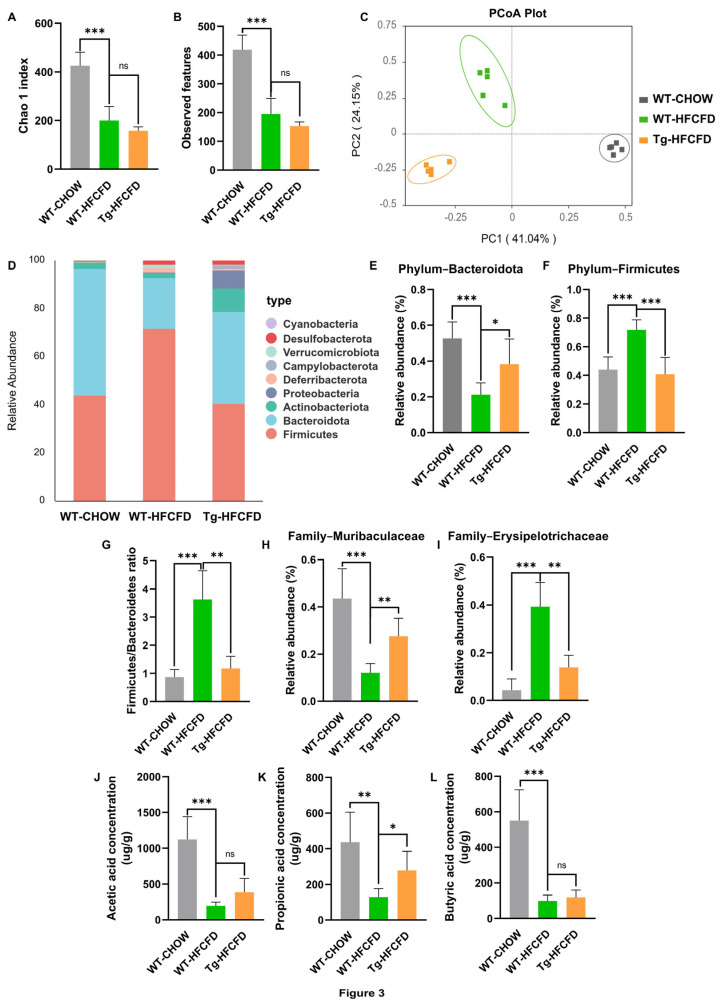
ApoA-I modulates gut microbiota composition and increases PPA level in MASLD mice. Microbial DNA from faecal samples of WT-CHOW, WT-HFCFD, and Tg-HFCFD mice was subjected to 16S rRNA sequencing. (**A**,**B**) Alpha diversity was evaluated using the Chao1 index and observed feature count. (**C**) Beta diversity visualised via principal coordinate analysis (PCoA) based on Bray–Curtis dissimilarity. (**D**) Phylum-level taxonomic composition of the gut microbiota. Relative abundance of (**E**) Bacteroidota and (**F**) Firmicutes at the phylum level. (**G**) Firmicutes to Bacteroidota ratio. Relative abundance of (**H**) Muribaculaceae and (**I**) Erysipelotrichaceae at the family level. Quantification of key faecal short-chain fatty acids, including (**J**) acetic acid, (**K**) propionic acid, and (**L**) butyric acid. Groups are color-coded: WT-CHOW (gray), WT-HFCFD (green), and Tg-HFCFD (orange). Corresponding group labels are shown on the x-axis. Data are expressed as mean ± SEM; *n* = 5 mice per group. * *p* < 0.05, ** *p* < 0.01, *** *p* < 0.001; ns represents no significant difference.

To characterise alterations in the intestinal flora induced by apoA-I, we conducted 16S rRNA gene sequencing on faecal samples obtained from WT-CHOW, WT-HFCFD, and Tg-HFCFD mice. Principal coordinate analysis clearly revealed segregation in the composition of the microbial communities across the three groups (Figure 3C), indicating that both diet and genotype significantly shaped the structure of the gut microbiota. Compared with WT-CHOW, HFCFD feeding significantly reduced alpha diversity, as assessed by the Chao index and observed features; however, compared with WT-HFCFD mice, apoA-I overexpression did not significantly alter microbial richness (Figure 3A,B).

We next examined specific taxonomic shifts. Analysis at the phylum level revealed that HFCFD induced a typical dysbiotic pattern characterised by a marked reduction in the abundance of Bacteroidota and a rise in the abundance of Firmicutes, thereby increasing the Firmicutes/Bacteroidota ratio, a hallmark of MASLD-associated dysbiosis [24]. This shift was notably reversed in the Tg-HFCFD mice (Figure 3D–G). Furthermore, at the family level, HFCFD feeding led to a reduction in Muribaculaceae, which is known as a key SCFA-producing family [25]. Concurrently, we observed an increase in Erysipelotrichaceae, a bacterial family that has been associated with steatosis and metabolic syndrome [26]. ApoA-I overexpression significantly counteracted these changes (Figure 3H,I).

Given that Muribaculaceae is a proficient producer of SCFAs, and considering the established importance of these metabolites in regulating glucose and lipid metabolism [12,13,16], we hypothesised that apoA-I may improve MASLD by increasing SCFA production. Consequently, we quantified the faecal SCFA levels. As expected, compared with CHOW-fed WT mice, mice on the HFCFD regimen exhibited substantial decreases in acetic, propionic, and butyric acid concentrations. Notably, the overexpression of apoA-I led to a specific and marked increase in PPA levels among HFCFD-fed mice (Figure 3J–L).

Collectively, these findings demonstrate that apoA-I remodels the gut microbiota in MASLD mice, attenuating diet-induced dysbiosis and specifically increasing the production of PPA, which may underlie its hepatoprotective mechanism.

### 2.4. PPA-Mediated Reduction in Lipid Accumulation Involves Enhanced Lipolysis and Fatty Acid Oxidation in HepG2 Cells

**Figure 4:**
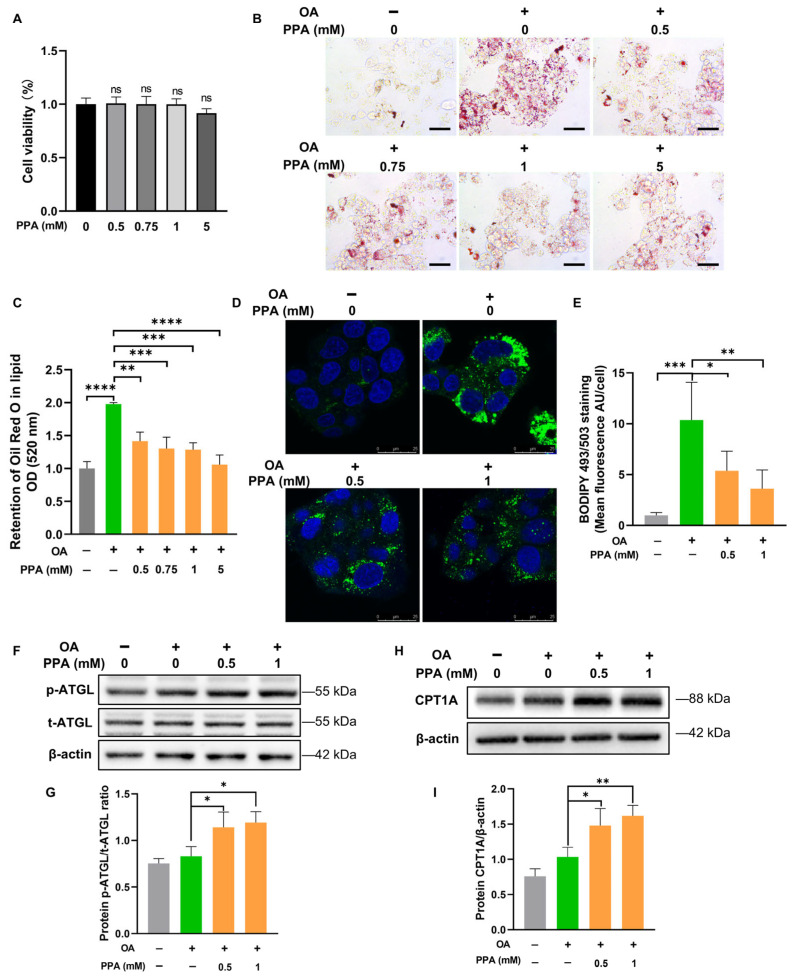
PPA-mediated reduction in lipid accumulation involves enhanced lipolysis and fatty acid oxidation in HepG2 cells. (**A**) Following 24 h incubation with 500 μM oleic acid (OA) or bovine serum albumin (BSA), HepG2 cells were exposed to PPA at concentrations of 0, 0.5, 0.75, 1, or 5 mM for an additional 48 h. Cell viability was determined via the CCK-8 assay. (**B**) Oil Red O staining images illustrate lipid droplets (scale bar = 200 μm). (**C**) Quantitative assessment of lipid content from Oil Red O staining. (**D**) Fluorescence images of BODIPY 493/503-stained lipid droplets (scale bar = 25 μm). (**E**) Mean fluorescence intensity quantification of BODIPY 493/503 staining. (**F**,**G**) Western blot analysis for detecting phosphorylated ATGL (p-ATGL) and total ATGL (t-ATGL). (**H**,**I**) CPT1A expression levels were examined by Western blotting. Colors represent the following groups: control (gray), OA alone (green), OA plus PPA at the indicated concentrations (orange). Group labels are displayed on the x-axis. Data represent mean ± SEM; *n* = 3. * *p* < 0.05, ** *p* < 0.01, *** *p* < 0.001, **** *p* < 0.0001; ns represents no significant difference.

To clarify how PPA influences hepatic lipid metabolism and explore its underlying mechanism, we investigated its effect on OA-induced lipid accumulation in HepG2 cells. Staining with Oil Red O revealed that at concentrations of 0.5 mM and greater, PPA effectively reduced lipid accumulation without compromising cell viability (Figure 4A–C). On the basis of these results, 0.5 mM and 1 mM PPA were selected for all subsequent experiments. Moreover, this concentration range effectively elicits biological activity in vitro and is well-established for investigating the functional roles and molecular mechanisms of PPA [27]. Consistently, BODIPY 493/503 staining further confirmed a marked decrease in both the quantity and size of lipid droplets in OA-loaded HepG2 cells following PPA treatment (Figure 4D,E). Given that lipid droplet catabolism is primarily regulated by lipolysis, we hypothesised that PPA may stimulate this catabolic pathway. To test this hypothesis, we evaluated the activity of adipose triglyceride lipase (ATGL), the primary regulatory enzyme that catalyses the first stage of triglyceride hydrolysis [28]. Western blot analysis demonstrated that PPA treatment significantly increased ATGL phosphorylation in OA-treated cells (Figure 4F,G), indicating the activation of intracellular lipolysis.

We next examined whether the fatty acids released via lipolysis were subsequently involved in oxidative metabolism. Accordingly, we examined the expression of carnitine palmitoyltransferase 1A (CPT1A), a mitochondrial enzyme essential for fatty acid β-oxidation [29]. Notably, PPA treatment robustly upregulated CPT1A expression (Figure 4H,I), suggesting a concomitant increase in fatty acid oxidation.

In summary, these results demonstrate that PPA mitigates hepatocellular lipid accumulation. This effect is associated with enhanced ATGL-mediated lipolysis and the promotion of fatty acid oxidation.

### 2.5. PPA Promotes ATGL-Mediated Lipolysis via GPR43–Ca^2^⁺–CAMKII Signalling Activation in HepG2 Cells

**Figure 5:**
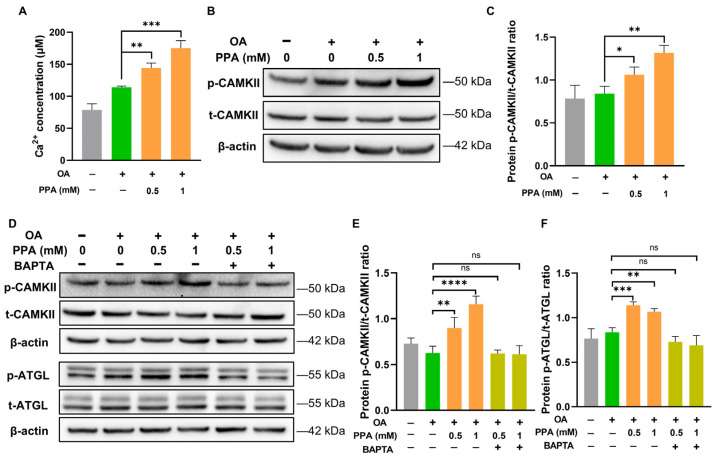
PPA promotes activation of CAMKII and ATGL in a Ca^2^⁺-dependent manner in OA-overloaded HepG2 cells. (**A**) After a 24 h incubation with 500 μM OA or BSA, HepG2 cells were exposed to 0.5 or 1 mM PPA for 48 h. Cytosolic Ca^2+^ levels were measured. (**B**,**C**) Western blot analysis for detecting phosphorylated CAMKII (p-CAMKII) and total CAMKII (t-CAMKII). (**D**–**F**) HepG2 cells pretreated with OA or BSA for 24 h were co-incubated with PPA (0.5 or 1 mM) in the presence or absence of 10 μM BAPTA. Protein extracts were then assessed for p-CAMKII, t-CAMKII, p-ATGL, and t-ATGL via Western blotting. Groups are denoted by color: control (gray), OA alone (green), OA + PPA (orange), and OA + PPA + BAPTA (lime green). PPA concentrations are as indicated. All groups are labeled on the x-axis. Data are expressed as mean ± SEM; *n* = 3. * *p* < 0.05, ** *p* < 0.01, *** *p* < 0.001, **** *p* < 0.0001; ns represents no significant difference.

Previous studies have shown that Ca^2^⁺–CaMKII signalling mediates ATGL phosphorylation and hepatic lipolysis [30,31]. Therefore, we evaluated alterations in intracellular Ca^2+^ levels and CAMKII phosphorylation in OA-overloaded HepG2 cells exposed to PPA. We observed a marked increase in intracellular Ca^2^⁺ levels and phosphorylated CAMKII in OA-overloaded HepG2 cells stimulated with 0.5 mM and 1 mM PPA (Figure 5A–C). To further investigate the role of Ca^2+^ in CAMKII and ATGL activation, we applied BAPTA, a calcium chelator, to sequester intracellular Ca^2+^. BAPTA treatment in OA-exposed HepG2 cells abolished the PPA-induced responses, which was supported by decreases in CAMKII and ATGL activity (Figure 5D–F). These results indicate that PPA promotes CAMKII and ATGL activation in a Ca^2+^-dependent manner in OA-overloaded HepG2 cells.

**Figure 6:**
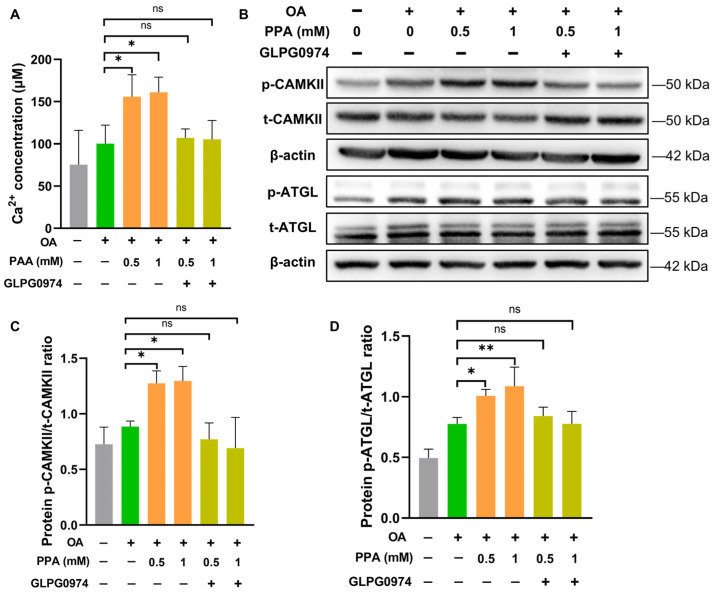
GPR43 is involved in PPA-induced activation of the Ca^2^⁺–CAMKII–ATGL pathway in HepG2 cells. (**A**) After a 24 h incubation with 500 μM OA or BSA, HepG2 cells were exposed to 0.5 or 1 mM PPA with or without 1 μM GLPG0974, after which cytosolic Ca^2^⁺ levels were measured. (**B**–**D**) Protein extracts were assessed for p-CAMKII, t-CAMKII, p-ATGL and t-ATGL via Western blotting. Groups are denoted by color: control (gray), OA alone (green), OA + PPA (orange), and OA + PPA + GLPG0974 (lime green). PPA concentrations are as indicated. All groups are labeled on the x-axis. Data represent mean ± SEM; *n* = 3. * *p* < 0.05, ** *p* < 0.01; ns represents no significant difference.

G-protein-coupled receptors 41 and 43 (GPR41, GPR43) function as the primary receptors for SCFAs and are involved in regulating lipid metabolism in the liver [14,16,32]. Upon activation by SCFAs, GPR43, which is expressed in the liver, triggers the Gαq-phospholipase C (PLC)-inositol trisphosphate (IP_3_) pathway, leading to increased intracellular Ca^2^⁺ release [33]. We therefore hypothesised that GPR43 participates in PPA-induced activation of the Ca^2^⁺–CAMKII–ATGL cascade. To test this hypothesis, we inhibited GPR43 using the specific antagonist GLPG0974. The results showed that GLPG0974 significantly attenuated the PPA-induced increase in the level of intracellular Ca^2^⁺ (Figure 6A), as well as the activation of CAMKII and ATGL (Figure 6B–D). Collectively, these data support the potential involvement of GPR43 in PPA-induced activation of the Ca^2^⁺–CAMKII–ATGL signalling axis in OA-overloaded HepG2 cells.

### 2.6. PPA Supplementation Ameliorates MASLD by Activating the CAMKII-ATGL Lipolysis Axis and Enhancing Mitochondrial β-Oxidation

**Figure 7:**
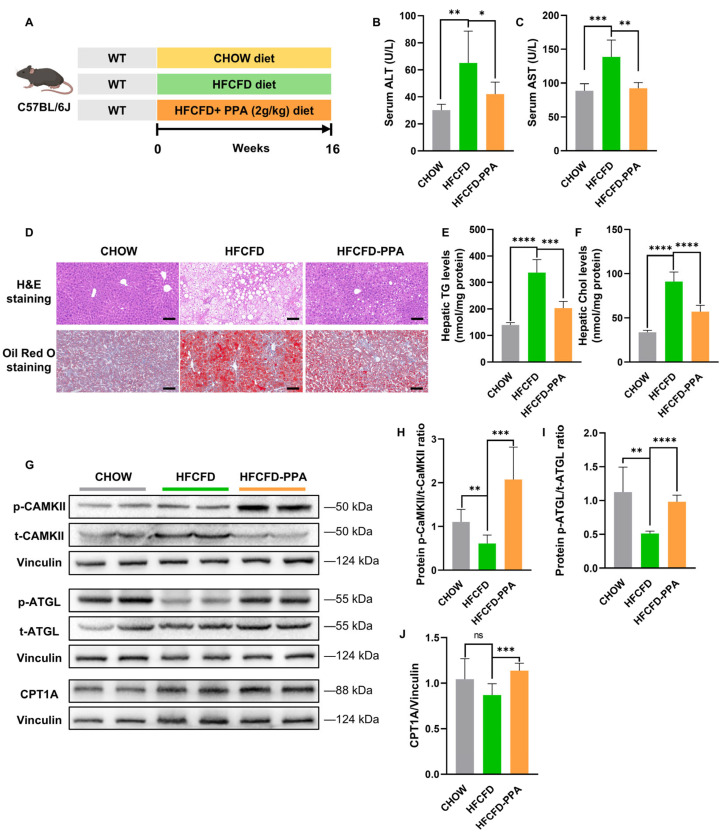
PPA supplementation ameliorates MASLD by activating the CAMKII-ATGL lipolysis axis and enhancing mitochondrial β-oxidation. (**A**) Male WT and apoA-I Tg mice, aged six weeks, were maintained on either a control chow diet, an HFCFD or HFCFD supplemented with 2 g/kg PPA for 16 weeks. Serum concentrations of (**B**) ALT and (**C**) AST were measured. (**D**) Liver sections were stained via H&E and Oil Red O (scale bar = 100 μm). Levels of (**E**) hepatic TG and (**F**) Chol were evaluated. (**G**–**J**) Liver tissue proteins were extracted and analysed for p-CAMKII, t-CAMKII, p-ATGL, t-ATGL and CPT1A via Western blotting. Groups are color-coded: CHOW (gray), HFCFD (green), and HFCFD-PPA (orange). Corresponding group labels are shown on the x-axis. Data represent mean ± SEM; *n* = 6 mice per group. * *p* < 0.05, ** *p* < 0.01, *** *p* < 0.001, **** *p* < 0.0001; ns represents no significant difference.

To evaluate whether PPA has therapeutic value for MASLD, 6-week-old male WT mice were assigned to three groups. Each group received one of three dietary regimens, namely, a chow diet, a HFCFD or a HFCFD supplemented with 2 g/kg PPA for 16 weeks (Figure 7A). Compared with the HFCFD-fed MASLD mice, the mice receiving PPA supplementation had notably lower serum concentrations of ALT and AST (Figure 7B,C; Table A3), indicating that PPA improved liver function. Additionally, examination of liver sections using H&E and Oil Red O staining confirmed that PPA supplementation mitigated HFCFD-induced hepatic steatosis and inflammatory infiltration (Figure 7D; Figure S2L–O). Consistent with these findings, hepatic TG and Chol levels, which were elevated in the HFCFD-fed mice, markedly decreased in the HFCFD-PPA-fed mice (Figure 7E,F). These findings collectively indicate that PPA exerts protective effects against MASLD progression by ameliorating hepatic lipid accumulation.

Furthermore, we investigated whether the Ca^2+^–CAMKII–ATGL signalling pathway contributes to the PPA-mediated amelioration of MASLD. Compared with mice in the HFCFD group, mice in the PPA supplementation group exhibited increased phosphorylation of CAMKII and ATGL (Figure 7G–I). Concurrently, we observed upregulation of CPT1A in the HFCFD-PPA-fed mice (Figure 7G,J). Taken together, these results demonstrate that PPA attenuates MASLD by promoting hepatic lipolysis through the activation of CAMKII and ATGL, and by enhancing hepatic mitochondrial β-oxidation.

## 3. Discussion

**Figure 8:**
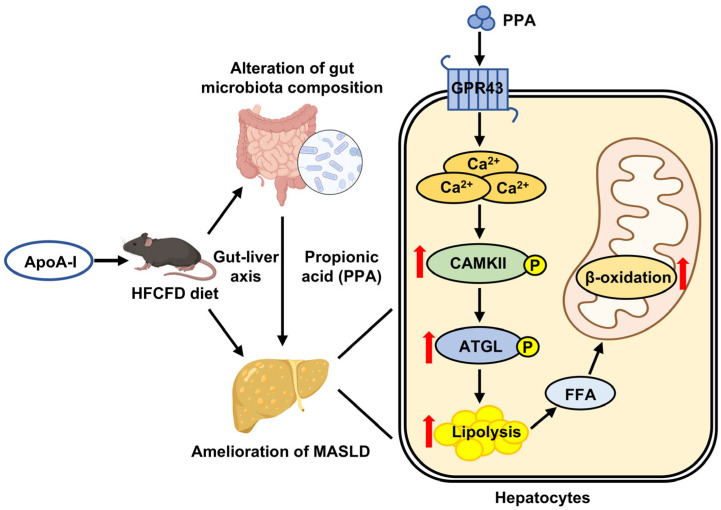
Schematic representation of the mechanism by which apoA-I ameliorates HFCFD induced MASLD mice. ApoA-I overexpression restores gut microbiota dysbiosis and elevates PPA levels, thereby ameliorating MASLD through stimulation of hepatic lipolysis and fatty acid oxidation. Black arrows denote the direction of protein regulation or physiological changes, whereas red arrows represent up-regulation of protein expression or enhancement of biological processes.

Our study reveals a previously unrecognised mechanism through which apoA-I alleviates MASLD in a gut microbiota-dependent manner. We demonstrated that apoA-I restored intestinal homeostasis, enriched the SCFA-producing family Muribaculaceae, and specifically elevated PPA levels. Functional validation in both HFCFD-fed mice and OA-treated HepG2 cells confirmed that PPA recapitulates the hepatoprotective effects of apoA-I. Mechanistically, our data suggest that PPA-induced hepatoprotection is associated with the activation of the GPR43–Ca^2^⁺–CaMKII–ATGL signalling cascade, promoting intracellular lipolysis and attenuating hepatic steatosis. These results identify PPA as a critical microbe-derived mediator of the benefits apoA-I and suggest that the apoA-I–gut microbiota–PPA axis is a novel therapeutic target for MASLD (Figure 8).

ApoA-I, the primary protein component of HDL, is predominantly produced in hepatic and intestinal tissues [34]. In concert with ATP-binding cassette transporter A1 (ABCA1), apoA-I facilitates reverse cholesterol transport (RCT), thereby enabling the efflux of extra cholesterol from peripheral tissues to the liver for subsequent elimination [35,36]. Building upon previous research [19,20,21], we confirmed that apoA-I alleviated HFCFD-induced MASLD in vivo. While several synthetic peptides designed to mimic apoA-I function (e.g., D-4F, L-4F and CSL-112) have been developed, their clinical application remains limited because of poor bioavailability and transient therapeutic effects [37,38,39,40]. Consequently, elucidating the underlying mechanisms by which apoA-I ameliorates MASLD is imperative.

Growing research underscores the critical involvement of intestinal flora imbalance and metabolite changes in MASLD pathogenesis via the gut–liver axis [41,42,43,44]. To model metabolic disturbances and intestinal microbial disruption, the mice in this study were maintained on an HFCFD. This dietary intervention is known to promote hepatic lipid accumulation, inflammatory infiltration [45], insulin resistance [19], and significant alterations in the gut microbiota composition [46]. Consistent with these established effects, HFCFD feeding induced a classic dysbiotic profile, which was effectively reversed by apoA-I overexpression. To functionally assess whether the intestinal microbiota plays a causal role in the hepatoprotective effects of apoA-I, we transplanted the faecal microbiota transplantation from HFCFD-fed apoA-I Tg mice into HFCFD-fed WT mice. This intervention significantly ameliorated MASLD pathology. Conversely, ABX-mediated gut microbiota depletion abolished the hepatoprotective effects of apoA-I. These findings demonstrated that the gut microbiota is essential for apoA-I-mediated protection against MASLD.

In addition to regulating cholesterol transport, apoA-I has intestinal anti-inflammatory effects [47,48]. Intestinal inflammation, which is a known feature of MASLD [49] and contributes to its progression [50], was clearly observed in our HFCFD-fed mice. This was evidenced by shortened colon length (Figure S3A,B), inflammatory cell infiltration, goblet cell depletion, and crypt damage (Figure S3C), along with elevated serum levels of tumour necrosis factor-α (TNF-α) and interleukin-1β (IL-1β) (Figure S3D,E). Notably, apoA-I overexpression effectively mitigated these inflammatory changes. Given that intestinal inflammation is known to contribute to gut dysbiosis [51], the ability of apoA-I to reduce inflammation may partly explain its ability to restore microbial homeostasis.

This microbial restructuring was characterised by specific and functional shifts in bacterial populations. ApoA-I overexpression specifically attenuated the HFCFD-induced expansion of Erysipelotrichaceae, a bacterial family linked to steatosis and metabolic syndrome [26], while concurrently enriching Muribaculaceae, a key SCFA-producing family [25]. Given the crucial role of SCFAs in modulating hepatic lipid metabolism and MASLD progression [52], we quantified faecal SCFA levels and observed a selective increase in the microbial metabolite PPA. The hepatoprotective role of PPA was further confirmed by direct supplementation, which demonstrated that PPA alone was sufficient to alleviate MASLD and reduce hepatic lipid accumulation. The supplemented dose (2 g/kg diet) was selected to align with effective levels reported in prior studies and was well tolerated, with no adverse effects on food intake or signs of overt toxicity [13,53].

Although the beneficial effects of SCFAs on hepatic lipid metabolism have been established, our study revealed a novel mechanism. Previous studies have demonstrated that PPA ameliorates steatosis primarily by inhibiting lipogenesis [12] and suppressing gluconeogenesis via the GPR43/AMPK pathway [54], whereas acetic acid also acts through hepatic GPR43 [14]. Beyond these previously described pathways, our data reveal a complementary mechanism, whereby PPA is associated with the activation of a GPR43–Ca^2^–CaMKII–ATGL cascade in steatotic hepatocytes, suggesting a potential role in stimulating intracellular lipolysis. Thus, PPA appears to promote lipid clearance not only by suppressing lipogenesis but also by enhancing lipolysis. In support of this view, PPA supplementation in MASLD mice suppressed lipogenic gene expression (Figure S6) and significantly elevated serum non-esterified fatty acid (NEFA) levels (Figure S4). Furthermore, PPA appeared to more potently stimulate lipolysis in steatotic HepG2 cells (Figure S7). Together, these findings suggest that enhanced hepatic lipolysis plays a key role in mediating the hepatoprotective effects of PPA.

Mechanistically, intracellular Ca^2^⁺ signalling is critically involved in the regulation of hepatic lipolysis. Previous studies have demonstrated that elevated cytosolic Ca^2^⁺ levels activate CaMKII and ATGL, thereby promoting lipolysis and mitochondrial fatty acid oxidation [30,31]. In line with these findings, our study revealed that PPA treatment increased intracellular Ca^2^⁺ concentrations, increased CaMKII and ATGL activation, and improved lipid metabolism. These effects were abolished by the calcium chelator BAPTA, underscoring the indispensability of Ca^2^⁺ signalling.

SCFAs are known to exert some of their effects through receptors such as GPR43, which has been implicated in hepatic lipid metabolism [55]. In our study, GPR43 expression was detectable in HepG2 cells and appeared to be influenced by PPA treatment (Figure S5). Furthermore, the PPA-induced increase in cytosolic Ca^2^⁺ and subsequent activation of CaMKII and ATGL were attenuated by the GPR43 antagonist GLPG0974. Although these pharmacological findings indicate that GPR43 participates in the Ca^2^⁺–CaMKII–ATGL pathway, further genetic studies are needed to definitively establish its role in PPA-mediated lipolysis in steatotic hepatocytes. Furthermore, our in vitro experiments were conducted in HepG2 cells, a hepatoma cell line that may not fully recapitulate the metabolic behaviour of primary hepatocytes. Despite these limitations, evidence from both in vivo and in vitro studies provides a coherent framework supporting the importance of the apoA-I–microbiota–PPA pathway in ameliorating MASLD and highlights its potential as a therapeutic target.

## 4. Materials and Methods

### 4.1. Animals

All experimental protocols involving animals complied with the ethical standards authorised by the Animal Ethics Committee of Capital Medical University (Approval No. AEEI-2025-1105). Male wild-type (WT) mice on a C57BL/6J background were provided by Vital River Laboratory (Beijing, China). Transgenic mice expressing human apolipoprotein A-I (apoA-I Tg; Strain: 001927) were obtained from The Jackson Laboratory. In this study, male mice of both genotypes, aged six weeks, were housed under specific pathogen-free (SPF) conditions with unrestricted access to food and drinking water.

#### 4.1.1. Animal Study 1: Effect of ApoA-I on MASLD Development

Male C57BL/6J WT and apoA-I Tg mice were evenly distributed into three experimental cohorts (n = 5 per group). The WT-CHOW group was maintained on a standard chow diet. The mice in the WT-HFCFD and Tg-HFCFD groups were fed a diet high in fat (40%), cholesterol (2%), and fructose (20%) (HFCFD; #D09100310; Xietong Biology, Yangzhou, China). All dietary regimens were provided ad libitum for a duration of 16 weeks.

#### 4.1.2. Animal Study 2: Role of the Gut Microbiota in ApoA-I-Mediated Amelioration of MASLD

Male C57BL/6J WT and apoA-I Tg mice were randomly allocated into five experimental groups (n = 5 per group). The mice assigned to the WT-CHOW group received a chow diet for the entire experimental period. The other four groups were provided with an HFCFD regimen that lasted 16 weeks. After the initial 8-week feeding period was completed, HFCFD-fed WT mice underwent faecal microbiota transplantation (FMT) from HFCFD-fed apoA-I Tg mice (designated the WT-HFCFD-FMT group). Concurrently, HFCFD-fed apoA-I Tg mice were given an antibiotic cocktail (ABX) to deplete the gut microbiota (designated the Tg-HFCFD-ABX group).

#### 4.1.3. Animal Study 3: Effect of Propionic Acid on MASLD Progression

Male C57BL/6J WT mice were randomly allocated into three groups (n = 6 per group): a standard chow diet (CHOW), HFCFD alone (HFCFD), or HFCFD supplemented with 2 g/kg propionic acid (PPA; #HY-W020017; MedChemExpress (MCE), Monmouth Junction, NJ, USA) (HFCFD-PPA). The study lasted 16 weeks. Body weights were recorded every two weeks, and food intake was strictly monitored to maintain consistent dietary consumption.

### 4.2. 16S rRNA Gene Sequencing and Microbiome Analysis

At the end of Animal Study 1, freshly obtained faeces samples from WT-CHOW, WT-HFCFD, and Tg-HFCFD mice were immediately flash-frozen in liquid nitrogen and preserved at −80 °C.

DNA extraction was performed, followed by PCR amplification of the V3–V4 hypervariable region of the bacterial 16S rRNA gene with the primer pair 341F/806R. The resulting amplicon libraries were subjected to paired-end sequencing on an Illumina NovaSeq 6000 system (Novogene, Beijing, China). Sequence data processing was conducted in QIIME2 (v2020.2): primers were removed with cutadapt (v2.8), and sequences were denoised and filtered for chimaeras using DADA2 to generate amplicon sequence variants (ASVs). Libraries containing fewer than 8000 quality-filtered sequences were discarded. The retained samples were rarefied to an even depth of 8500 sequences per sample. Taxonomic classification was carried out with a pretrained naïve Bayes classifier (silva-132-99-nb-classifier.qza) referencing the SILVA 132 database at 99% identity. Alpha diversity was calculated using the Chao1 and observed features metrics. Moreover, beta diversity was quantified with the Bray–Curtis distance, displayed via principal coordinate analysis (PCoA), and group differences were statistically tested using ANOSIM. The differential abundance of bacterial taxa across groups was examined with the Mann–Whitney U test. Raw sequencing reads have been deposited in the NCBI SRA under BioProject accession number PRJNA1377218.

### 4.3. Short-Chain Fatty Acid (SCFA) Analysis by LC–MS/MS

The concentrations of faecal short-chain fatty acids (SCFAs) were determined via an established LC–MS/MS method. Sample analysis was carried out on a TSQ Altis™ mass spectrometer coupled to a Vanquish™ Flex UHPLC system (Thermo Fisher Scientific, Waltham, MA, USA). Separation was achieved with a Waters ACQUITY UPLC BEH C18 column (2.1 × 100 mm, 1.7 μm; Waters Corporation, Milford, MA, USA) maintained at 40 °C, employing a gradient elution of 10 mM aqueous ammonium acetate (Sigma-Aldrich, St. Louis, MO, USA) and a mixture of acetonitrile and isopropanol (1:1; Sigma-Aldrich, St. Louis, MO, USA). Detection was performed in negative ionisation mode using multiple reaction monitoring (MRM). Quantitation was based on external calibration curves corrected by isotopically labelled internal standards specific to each SCFA. Method validation confirmed acceptable precision (intra- and interday RSD ≤ 15%), accuracy (85–115% recovery), and stability (RSD ≤ 15% over 24 h at 4 °C), supporting the reliable quantification of all the analytes.

### 4.4. Antibiotic Cocktail (ABX) Treatment

To eliminate the gut microbiota, the mice were subjected to a two-stage antibiotic treatment. During the initial intensive phase (the first week), each mouse received a daily oral dose of a 200 μL antibiotic mixture containing neomycin sulphate (200 mg/kg; #N6386; Sigma-Aldrich, St. Louis, MO, USA), metronidazole (200 mg/kg; #A114755; Ambeed, Buffalo Grove, IL, USA), vancomycin (100 mg/kg; #A810922; Ambeed, Buffalo Grove, IL, USA), and ampicillin (200 mg/kg; #A307483; Ambeed, Buffalo Grove, IL, USA). Subsequently, during the maintenance period spanning 8 weeks, the animals’ drinking water was supplemented with neomycin sulphate (1 g/L), metronidazole (1 g/L), ampicillin (1 g/L), and vancomycin (500 mg/L). This antibiotic-supplemented water was freshly made and changed every 48 h to ensure its effectiveness.

### 4.5. Faecal Microbiota Transplantation (FMT)

Recipient mice were pretreated with antibiotics for three days to eliminate their endogenous intestinal microbiota prior to FMT. Fresh faecal pellets from Tg-HFCFD donor mice were suspended in sterile PBS, and then subjected to centrifugation at 500× *g* for 5 min. The supernatant was collected under anaerobic conditions. Recipient mice (WT-HFCFD-FMT) received 200 μL of this faecal supernatant by oral gavage four times weekly for 8 weeks.

### 4.6. Liver Sample Collection and Morphological Analysis

After a 10 h fasting period with water provided ad libitum, the mice were euthanised following approved protocols. Livers were promptly excised. A consistent 1 cm × 1 cm section from each mouse was fixed in 4% paraformaldehyde (#G1101; Servicebio, Wuhan, China), processed for paraffin embedding, sectioned, and subjected to hematoxylin and eosin (H&E) staining for histological evaluation. For lipid analysis, fresh liver tissue was embedded in optimal cutting temperature (OCT) medium, rapidly frozen, and cryosectioned for Oil Red O staining. The residual hepatic tissue was snap-frozen in liquid nitrogen and maintained at −80 °C for subsequent analysis.

### 4.7. Histopathological Scoring and Quantitative Image Analysis

Hepatic steatosis, ballooning, and inflammation were graded on H&E-stained sections on the basis of established criteria (Supplementary Tables S1–S3). For each mouse, 5 random nonoverlapping fields (200×) were examined. Scoring was conducted independently by two pathologists who were blinded to group allocation, and the final scores represent the average of both assessments. To quantify steatosis, Oil Red O-stained liver sections were analysed using ImageJ (version 1.53; National Institutes of Health, Bethesda, MD, USA). The software was used to determine both the lipid-positive area and the total tissue area, and the proportion of lipid area was calculated as (lipid-positive area/total tissue area) × 100%.

### 4.8. Determination of Hepatic Triglyceride and Total Cholesterol Contents

Liver triglyceride (TG) and total cholesterol (TC) levels were quantified according to kit protocols (#E1025-105 and #E1026-105; Applygen Technologies, Beijing, China). Approximately 25 mg of frozen liver was homogenised in 500 µL of lysis buffer and clarified by centrifugation at 2000× *g* for 5 min. The resulting supernatant was split for protein quantification (BCA assay; #23227; Thermo Fisher Scientific, Waltham, MA, USA) and TG/TC assays. Reagent solutions (R1:R2 = 4:1) and standard curves (glycerol for TG, cholesterol for TC) were prepared. Ten microlitres from each sample was combined with 190 µL of working solution in a 96-well plate, followed by incubation at 37 °C for 15 min (TG assay) or 20 min (TC assay). The absorbance was recorded at 550 nm, and the TG/TC levels were normalised to the total protein content.

### 4.9. Biochemical Analysis

Blood samples were collected and centrifuged at 3000 rpm for 15 min to obtain serum. Alanine aminotransferase (ALT) and aspartate aminotransferase (AST) levels were determined with an automatic biochemical analyser (Rayto Life and Analytical Sciences, Shenzhen, China). Serum non-esterified fatty acids (NEFA) were measured using a commercial kit (#A042-2-1; Nanjing Jiancheng Bioengineering Institute, Nanjing, China). Serum levels of tumour necrosis factor-α (TNF-α) and interleukin-1β (IL-1β) were evaluated by ELISA (#JL10484 and #JL18442, respectively; Jianglai Biotechnology, Shanghai, China).

### 4.10. Cell Culture and Treatment

#### 4.10.1. HepG2 Cell Culture

The HepG2 human hepatoma cell line (obtained from ATCC) was maintained under standard conditions at 37 °C with 5% CO_2_. The culture medium consisted of high-glucose Dulbecco’s modified Eagle’s medium (DMEM) supplemented with 10% foetal bovine serum (FBS) and 1% penicillin–streptomycin.

#### 4.10.2. In Vitro MASLD Model Establishment and Treatment

To establish a MASLD cell model, HepG2 cells were initially incubated with 500 μM oleic acid (OA; #O1383; Sigma-Aldrich, St. Louis, MO, USA) or bovine serum albumin (BSA; as a control) for 24 h to induce lipid accumulation. The cells were subsequently treated with different doses of propionic acid (PPA; 0, 0.5, 0.75, 1, or 5 mM) for an additional 48 h period.

To investigate the role of cytosolic Ca^2^⁺ in the protective effects of PPA on MASLD in vitro, the calcium chelator BAPTA (10 μM; #HY-100168; MCE, Monmouth Junction, NJ, USA) was used to inhibit intracellular Ca^2^⁺ levels. Additionally, the free fatty acid receptor-2 (FFA2/GPR43) antagonist GLPG0974 (1 μM; #HY-12940; MCE, Monmouth Junction, NJ, USA) was used to explore the potential involvement of GPR43 signalling.

### 4.11. Cell Viability Assessment

A CCK-8 assay (#A311-01; Vazyme, Nanjing, China) was used to assess cell viability in accordance with the supplier’s guidelines.

### 4.12. Oil Red O Staining

The Oil Red O method was used to detect lipid accumulation. The cells were fixed for 15 min at room temperature using 4% paraformaldehyde (#G1101; Servicebio, Wuhan, China). Following the PBS washes, the samples were incubated with Oil Red O working solution (#O8010; Solarbio, Beijing, China) for 30 min in the dark. Stained lipid droplets were then examined under a microscope. For quantification, lipid droplets were extracted with 4% NP-40 solution (#N8030; Solarbio, Beijing, China), and the absorbance was read at 520 nm on a microplate reader.

### 4.13. BODIPY 493/503 Staining

Intracellular lipids were visualised using a Lipid Droplets Green Fluorescence Assay Kit (#C2053; Beyotime Biotechnology, Shanghai, China) as directed. Briefly, after being fixed with 4% paraformaldehyde and washed with PBS, the cells were exposed to staining solution (containing BODIPY 493/503 and Hoechst 33342) for 20 min in the dark. Following a final PBS wash, images were captured using a confocal microscope.

### 4.14. Intracellular Calcium Measurement

Cytosolic Ca^2^⁺ concentrations were determined using a calcium assay kit (#S1063S; Beyotime Biotechnology, Shanghai, China) in strict accordance with the provided protocol, and absorbance readings were taken at 575 nm.

### 4.15. Western Blot Analysis

Proteins from HepG2 cells or liver samples were extracted using lysis buffer supplemented with protease (#B14001; Selleck, Shanghai, China) and phosphatase (#B15001; Selleck, China) inhibitor cocktails. The protein concentration was quantified via a BCA assay (#23227; Thermo Fisher Scientific, Waltham, MA, USA).

Protein levels were evaluated by Western blotting using antibodies against phospho-CaMKII (#12716T; Cell Signaling Technology (CST), Danvers, MA, USA), CaMKII (#4436T; CST, Danvers, MA, USA), phospho-ATGL (#ab317611; Abcam, Cambridge, UK), ATGL (#55190-1-AP; Proteintech, Rosemont, IL, USA), β-actin (#60008-1-Ig; Proteintech, Rosemont, IL, USA), apoA-I (#14427-1-AP; Proteintech, Rosemont, IL, USA), GPR43 (#84544-1-RR; Proteintech, Rosemont, IL, USA) and vinculin (#26520-1-AP; Proteintech, Rosemont, IL, USA). The band intensity was analysed using ImageJ software (version 1.53; National Institutes of Health, Bethesda, MD, USA).

### 4.16. Colon Length Measurement and Histological Analysis

The colon was excised, and its length was recorded. Colon segments were histologically evaluated after fixation in 4% paraformaldehyde, paraffin embedding, and sectioning at a thickness of 5 μm. The sections were subsequently stained with H&E for microscopic observation.

### 4.17. Statistics

The results are expressed as the mean ± SEM. GraphPad Prism (version 9.0) software was used for all the statistical analyses, with *p* < 0.05 considered to indicate statistical significance. Differences between two groups were assessed using two-tailed Student’s *t* tests. Comparisons involving three or more groups were performed by one-way ANOVA, followed by Tukey’s post hoc test.

## 5. Conclusions

In summary, this study reveals a new pathway in which apoA-I modulates the gut microbiota composition and increases PPA levels to ameliorate MASLD. Our findings indicate the protective effects of PPA involve the GPR43–Ca^2^⁺–CaMKII–ATGL pathway, which promotes hepatic lipolysis and fatty acid oxidation. These results provide links among apoA-I, the gut microbiota, PPA and MASLD and thereby highlight the apoA-I–microbiota–PPA axis as a promising target for future therapeutic development.

## Data Availability

The original contributions presented in this study are included in the article/Supplementary Materials. Further inquiries can be directed to the corresponding author(s). Raw 16S rRNA sequencing data are publicly accessible in the NCBI Sequence Read Archive under BioProject ID PRJNA1377218.

## Supplementary Materials

The following supporting information can be downloaded at: https://www.mdpi.com/article/10.3390/ijms27010468/s1.

## Abbreviations

The following abbreviations are used in this manuscript:

16S rRNA	16S ribosomal RNA
ABCA1	ATP-binding cassette transporter A1
ABX	antibiotic cocktail
ALT	alanine transaminase
apoA-I	apolipoprotein A-I
AST	Aspartate transferase
ATGL	adipose triglyceride lipase
CaMKII	Ca^2^⁺/calmodulin-dependent protein kinase II
Chol	cholesterol
CPT1A	carnitine palmitoyltransferase 1A
FMT	faecal microbiota transplantation
GPR41	G-protein-coupled receptor 41
GPR43	G-protein-coupled receptor 43
HCC	hepatocellular carcinoma
H&E	hematoxylin and eosin
HDL	high-density lipoprotein
HFCFD	high-fat, high-cholesterol, high-fructose diet
IL-1β	interleukin-1β
MASLD	metabolic dysfunction-associated steatotic liver disease
MASH	metabolic dysfunction-associated steatohepatitis
NEFA	non-esterified fatty acid
OA	oleic acid
PCoA	principal coordinates analysis
PPA	propionic acid
RCT	reverse cholesterol transport
SCFA	short chain fatty acid
TG	triglyceride
Tg	transgenic
TNF-α	tumor necrosis factor-α
WT	wild-type

## Appendix A

**Table A1:** Serum biochemical parameters in mice of Animal study 1.

	WT-CHOW	WT-HFCFD	Tg-HFCFD
ALT (U/L)	29.66 ± 7.91	181.04 ± 113.10 *	62.97 ± 13.38 #
AST (U/L)	100.30 ± 22.50	316.89 ± 139.82 **	150.37 ± 35.68 #

Data are expressed as mean ± SEM, n = 5. * *p* < 0.05 and ** *p* < 0.01 compared with WT-CHOW group; # *p* < 0.05 compared with WT-HFCFD group.

**Table A2:** Serum biochemical parameters in mice of Animal study 2.

	WT-CHOW	WT-HFCFD	Tg-HFCFD	WT-HFCFD-FMT	Tg-HFCFD-ABX
ALT (U/L)	33.21 ± 4.27	182.67 ± 93.98 **	46.14 ± 29.97 ##	38.69 ± 3.81 ##	134.34 ± 76.01 $
AST (U/L)	126.75 ± 36.22	271.40 ± 91.47 **	137.03 ± 29.33 ##	125.25 ± 33.14 ##	210.51 ± 42.88 $$

Data are expressed as mean ± SEM, n = 5. ** *p* < 0.01 compared with WT-CHOW group; ## *p* < 0.01 compared with WT-HFCFD group; $ *p* < 0.05 and $$ *p* < 0.01 compared with Tg-HFCFD group.

**Table A3:** Serum biochemical parameters in mice of Animal study 3.

	CHOW	HFCFD	HFCFD-PPA
ALT (U/L)	30.16 ± 4.35	65.14 ± 23.55 **	42.03 ± 8.82 #
AST (U/L)	88.82 ± 10.26	138.97 ± 24.54 ***	92.34 ± 8.22 ##

Data are expressed as mean ± SEM, n = 6. ** *p* < 0.01 and *** *p* < 0.001 compared with CHOW group; # *p* < 0.05 and ## *p* < 0.01 compared with HFCFD group.
