# Supplementary material for: Gut Microbiota-Derived Propionic Acid Mediates ApoA-I-Induced Amelioration of MASLD via Activation of GPR43–Ca2+–CAMKII–ATGL Hepatic Lipolysis
Source: Int J Mol Sci. 2026 Jan 1;27(1):468. doi: 10.3390/ijms27010468 (PMC12787056; doi:10.3390/ijms27010468)
Supplement: Supplementary file 1 [file ijms-27-00468-s001.zip › ijms-4033482-supplementary.pdf]

Supplementary Materials

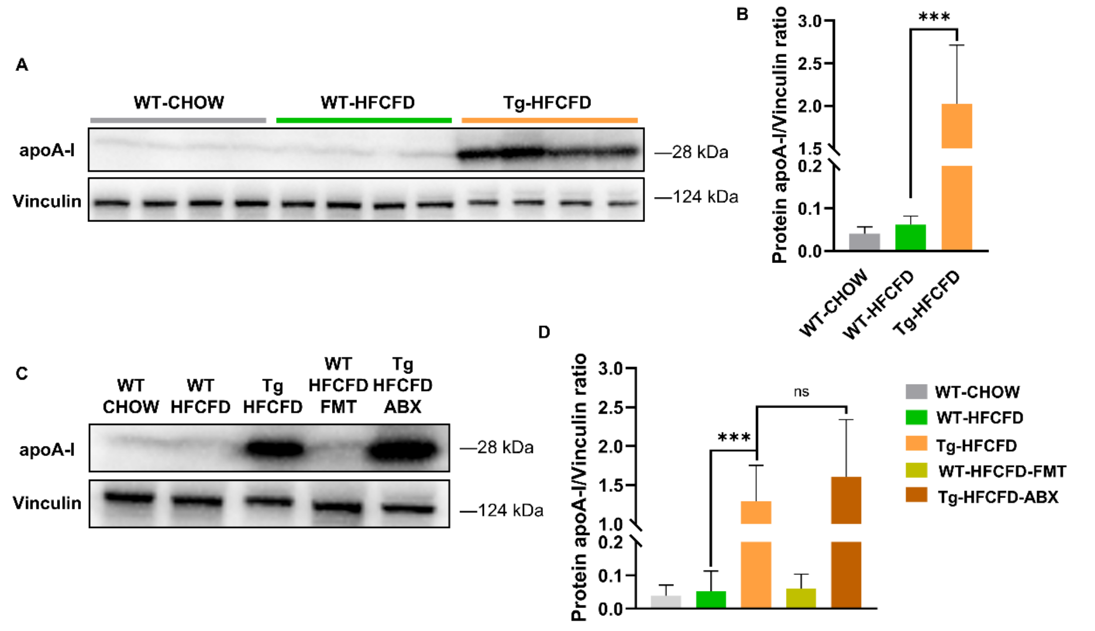

Figure S1. Hepatic apoA-I protein levels were assessed by western blotting. (A, B) Representative western blot (A) and quantitative analysis (B) of apoA-I expression in liver tissues from WT-CHOW, WT-HFCFD, and Tg-HFCFD mice (n = 5 mice per group). (C, D) Representative western blot (C) and quantitative analysis (D) of apoA-I expression in liver tissues from WT-CHOW, WT-HFCFD, Tg-HFCFD, WT-HFCFD-FMT and Tg-HFCFD-ABX mice (n = 5 mice per group). Data are presented as mean ± SEM. *** P<0.001.

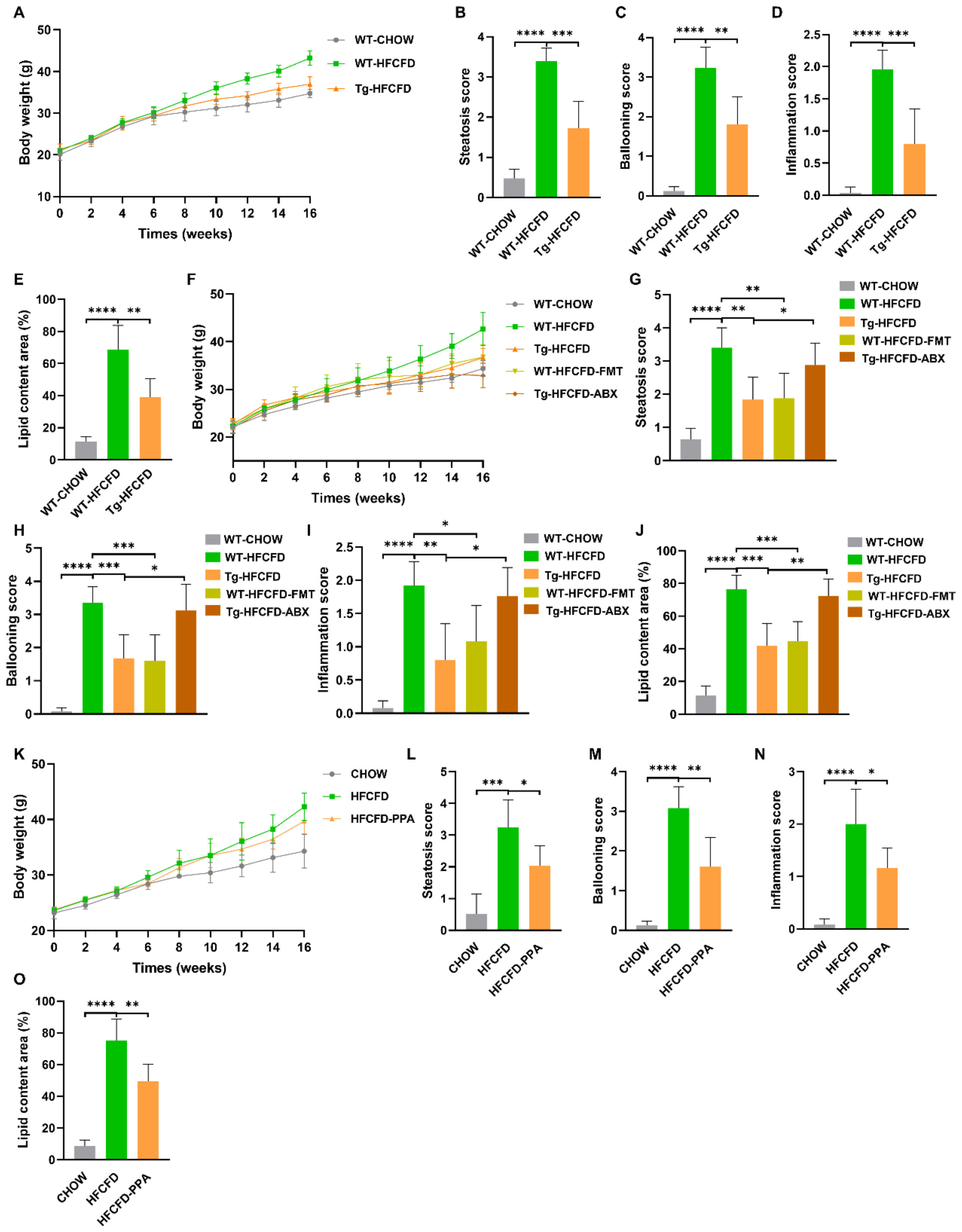

Figure S2. Body weight and quantitative assessment of liver histopathology in three animal studies. (A) Body weight changes during the 16-week period in Animal Study 1. (B-E) Liver histopathology was scored in Animal Study 1: (B) steatosis score, (C) ballooning score, (D) inflammation score based on H&E staining, and (E) hepatic lipid area was quantified from Oil Red O staining. (F) Body weight changes during the 16-week period in Animal Study 2. (G-J) Liver histopathology was scored in Animal Study 2: (G) steatosis score, (H) ballooning score, (I) inflammation score based on H&E staining, and (J) hepatic lipid area was quantified from Oil Red O staining. (K) Body weight changes during the 16-week period in Animal Study 3. (L-O) Liver histopathology was scored in Animal Study 3: (L) steatosis score, (M) ballooning score, (N) inflammation score based on H&E staining, and (O) hepatic lipid area was

quantified from Oil Red O staining. Data are presented as mean ± SEM. For Animal Study 1 and 2, n = 5 mice per group; for Animal Study 3, n = 6 mice per group. * P<0.05, ** P<0.01, *** P<0.001 and **** P<0.0001.

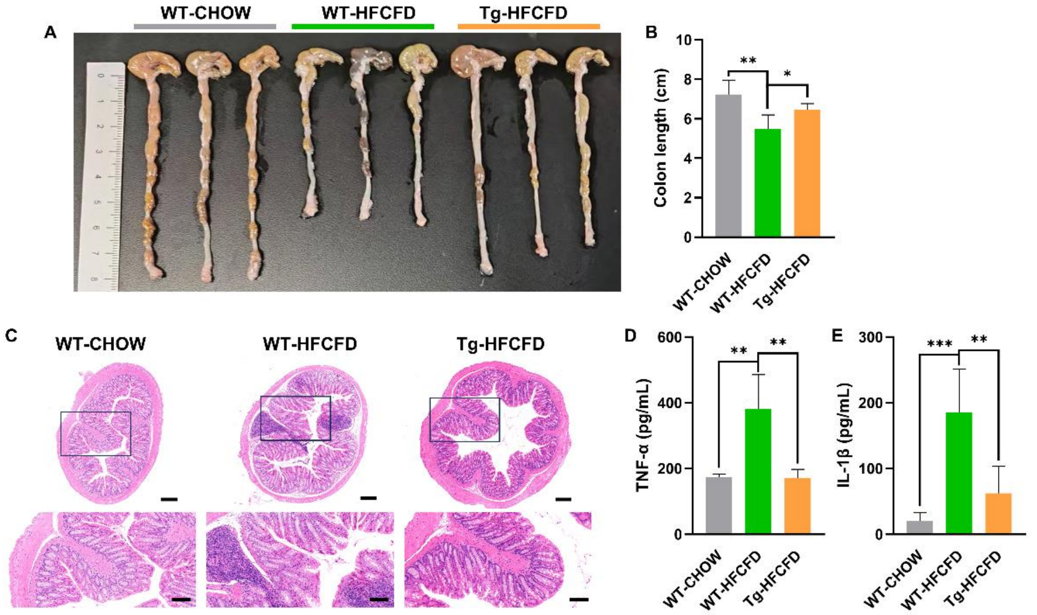

Figure S3. ApoA-I exhibits potential ameliorative effects on intestinal inflammation. (A) Representative colon images of the WT-CHOW, WT-HFCFD, and Tg-HFCFD mice. (B) Colon length was measured among the three groups of mice. (C) Colon tissue sections were examined by H&E staining (scale bar = 100 μm). (D, E) Serum levels of TNF-α and IL-1β were measured in the three groups. Data are presented as mean ± SEM; n = 5 mice per group. * P<0.05, ** P<0.01 and *** P<0.001.

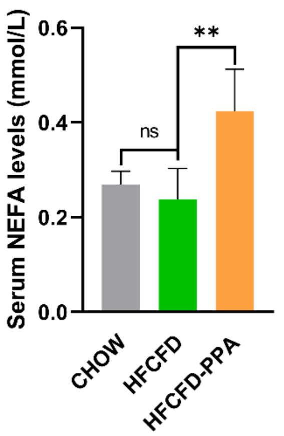

Figure S4. PPA supplementation increases serum levels of NEFAs in HFCFD-fed mice. Serum levels of NEFA in mice fed with CHOW, HFCFD or HFCFD-PPA were measured. Data are presented as mean ± SEM; n = 6 mice per group. ** P<0.01.

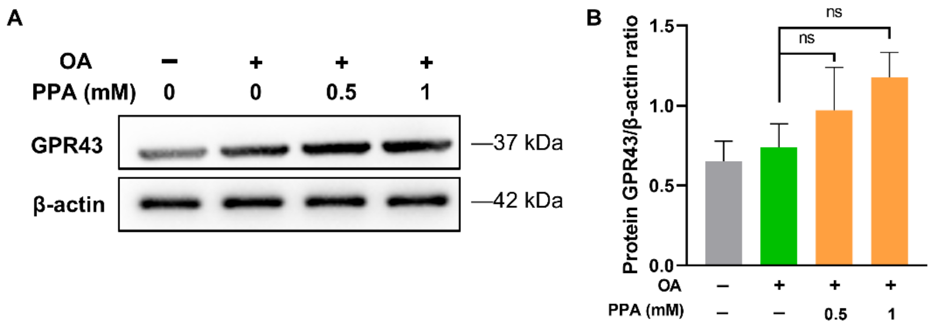

Figure S5. GPR43 expression in HepG2 cells following PPA treatment. (A) HepG2 cells were treated with BSA or 500 μM OA for 24 h, and then exposed to 0.5 or 1 mM PPA for 48 h. (B, C) Total cellular proteins were isolated and analyzed for GPR43 by Western blotting. Data are presented as mean ± SEM.

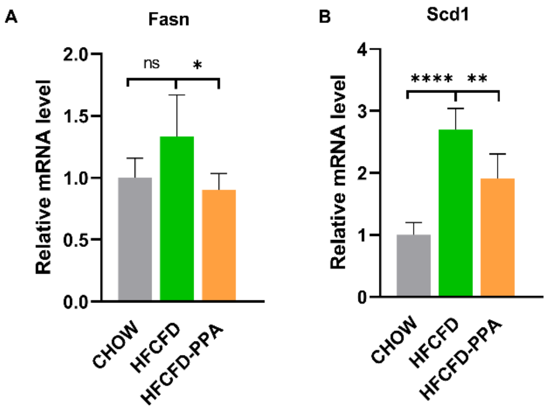

Figure S6. PPA supplementation downregulates hepatic lipogenic gene expression in HFCFD-fed mice. (A) mRNA expression of Fasn. (B) mRNA expression of Scd1. Data are presented as mean ± SEM; n = 5 mice per group. * $P < 0.05$, ** $P < 0.01$ and **** $P < 0.0001$.

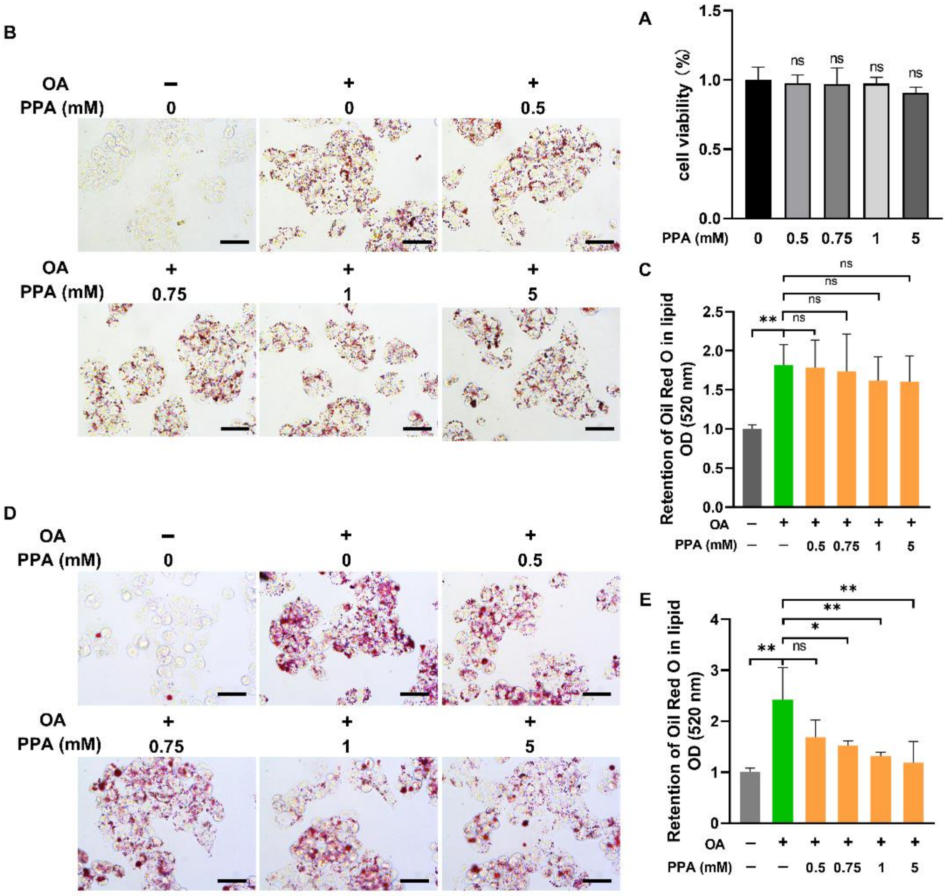

Figure S7. PPA may more strongly promote lipolysis than suppress lipogenesis in HepG2 cells. (A) HepG2 cells were treated with BSA for control, 500 μM OA, or OA combined with PPA at 0, 0.5, 0.75, 1, or 5 mM for 24 h. Cell viability was assessed using the CCK-8 assay. (B) Representative images of Oil Red O staining show lipid droplets in HepG2 cells (scale bar = 200 μm). (C) Quantification of Oil Red O staining. (D) HepG2 cells were treated with BSA or 500 μM OA for 24 h, followed by stimulation with PPA at 0, 0.5, 0.75, 1, and 5 mM for an additional 24 h. Representative images of Oil Red O staining show lipid droplets in HepG2 cells (scale bar = 200 μm). (E) Quantification of Oil Red O staining. Data are presented as mean ± SEM. * $P<0.05$ and ** $P<0.01$.

Table S1. Liver steatosis scoring criteria

Score	Criteria
0	No steatosis (<5%)
1	Minimal steatosis (5%–<25%)
2	Mild steatosis (25%–<50%)
3	Moderate steatosis (50%–<75%)
4	Severe steatosis (≥75%)

Table S2. Liver ballooning scoring criteria

Score	Criteria
0	No hepatocyte ballooning
1	Minimal ballooning (affecting<25% of hepatocytes)
2	Mild ballooning (affecting 25%–<50% of hepatocytes)

Score	Criteria
3	Moderate ballooning (affecting 50%–<75% of hepatocytes)
4	Severe ballooning (affecting ≥75% of hepatocytes)

Table S3. Liver inflammation scoring criteria

Score	Criteria
0	No inflammatory foci
1	1–3 inflammatory foci per 200×field
2	4–5 inflammatory foci per 200×field
3	>5 inflammatory foci (focal necrosis) per 200×field
4	Confluent necrosis involving >25% of the 200×field
